# Association between constipation and the development of asthma: a meta-analysis

**DOI:** 10.1186/s13223-022-00708-9

**Published:** 2022-08-08

**Authors:** Lu Liu, Xiangli Zhang, Zhengdong Jiang, Guizuo Wang, Hua Wu, Ruilin Chen, Yongqing Zhang, Manxiang Li, Shumei Yang

**Affiliations:** 1 Department of Respiratory and Critical Care Medicine, Shaanxi Provincial People’s Hospital, No. 256, West Youyi Road, Xi’an, Shaanxi 710068 People’s Republic of China; 2 Department of General Surgery, The First Affiliated Hospital of Xi’an Jiaotong University, No. 277, West Yanta Road, Xi’an, Shaanxi 710061 People’s Republic of China; 3 Department of Respiratory and Critical Care Medicine, The First Affiliated Hospital of Xi’an Jiaotong University, No. 277, West Yanta Road, Xi’an, Shaanxi 710061 People’s Republic of China

**Keywords:** Constipation, Wheezing, Asthma, Meta-analysis

## Abstract

**Background:**

Constipation has been hypothesized to be associated with the increased risk of wheezing or asthma. However, the relation remains a subject of debate. We conducted this meta-analysis to assess whether constipation influences the risk of wheezing/asthma.

**Methods:**

PubMed, Embase, and Web of Science were systematically searched for studies published between 1955 and January 2022. Two reviewers independently extracted data and assessed the quality of each study. Results were pooled using fixed-effects models or random-effects models as appropriate.

**Results:**

In total, 3 original articles with 178,661 participants, which met the criteria, were included in this meta-analysis. Constipation was associated with an increased risk of wheezing/asthma in later life (RR = 2.02, 95% CI = 1.24–3.29, P < 0.01).

**Conclusions:**

The meta-analysis suggests an association between constipation and the subsequent development of wheezing/asthma. Well-designed and highly standardized prospective studies that adequately address concerns for potential confounding factors are required to validate the risk identified in our current meta-analysis.

**Supplementary Information:**

The online version contains supplementary material available at 10.1186/s13223-022-00708-9.

## Background

Asthma is one of the most common chronic airway inflammatory diseases arising from not fully understood heterogenic gene-environment interactions [1], which has clearly shown to be a global public health problem affecting children and adults. It not only increases financial, social and psychological burdens, but also makes a harmful impact on quality of life [2]. Risk factors for asthma are diverse, including respiratory viral infections, air pollution, genetic factors, atopy and others [3–6].

Constipation is a common complaint that may be primary (idiopathic or functional) or associated with several disorders or medications. The median prevalence of constipation is 16% in adults and 3% in children [7], it is greater in non-white populations, institutionalized people, and women [8]. Although constipation rarely leads to life-threatening complications, it is a cause of physical and psychological distress for patients and their families, eventually impairing quality of life and leading to increased health insurance costs [9]. Stool stasis has long been considered to influence the microbiota and environment of gut, resulting in deleterious effects on mucosal immunity and intestinal motility [10]. The gut microbiota might exert important regulatory effects via the gut–lung axis [11–13]. Recently, several studies have shown the constipation might be linked to increased risk of the subsequent development of wheezing or asthma [14–16]. Constipation and asthma, seemingly disparate, conditions share several common pathogenetic features. An altered contractility and smooth muscle tone have been observed in both diseases [15–17]. However, other researchers think that wheezing symptoms are not associated with constipation after adjusting for the child’s exposure to infections and use of antibiotics [18].

The demonstration of a relation between constipation and the development of later wheezing/asthma, as well as a better understanding of the nature of this association, could have important implications for the prevention and treatment of asthma. To the best of our knowledge, no study has been published that has systematically reviewed the literature and synthesized the available evidence. Therefore, we performed a systematic review and meta-analysis of the existing evidence.

## Methods

The study was conducted according to the Preferred Reporting Items for Systematic Reviews and Meta-Analyses (PRISMA) guidelines.

### Search strategy

Two independent reviewers (LL and ZJ) systematically searched the PubMed, EMBASE, Web of Science to identify available studies published between 1955 and January 2022. The search terms used were constipation AND (wheezing OR asthma). A search strategy for each database was established with the help of a library expert of Xi’an Jiaotong University. The search details using PubMed, EMBASE and Web of science were shown in Additional file 1: Table S1. Additional studies were found by searching reference lists of identified articles.

### Inclusion and exclusion criteria

Studies were included in the meta-analysis that met the following criteria: (1) cohort study or case–control study; (2) diagnosis of constipation was made on the basis of one of the following: physician diagnosis of constipation, diagnostic code from the Health Insurance Research Database, or assessment questionnaire; (3) outcome of interest was wheezing/asthma (diagnosis was made on the basis of one of the following: physician diagnosis of wheezing/asthma, diagnostic code from the Health Insurance Research Database, a prescription for an anti-asthmatic medication including inhaled corticosteroids (ICSs), short-acting β-agonists, and systemic corticosteroids within 1 year, or assessment questionnaire); (4) measures of association [odds ratio (OR), the odds that an outcome will occur given a particular exposure, compared to the odds of the outcome occurring in the absence of that exposure, or relative risk (RR), the ratio of the risk of developing a disease among subjects with the risk factor to the risk of developing the disease among subjects without the risk factor, or hazard ratio (HR), hazard in the exposed groups divided by the hazard in the unexposed groups] and their 95% CI or data allowing for computation of summary measures are provided. Studies were excluded if they did not meet these inclusion criteria. Unpublished data were not considered. Study selection was achieved by two investigators (YZ and YS) independently and all the disagreements were resolved by discussion.

### Quality assessment

The quality of each study included in this study were independently assessed by two reviewers (ZJ and LL) according to the Newcastle–Ottawa Scale (NOS) [19]. We considered a study awarded 0–3, 4–6, or 7–9 as a low-quality, moderate-quality, or high-quality study, respectively. Discrepancies were resolved by consensus and discussion.

### Data extraction

Data were extracted by two independent reviewers (LL and XZ) using a standardized data collection form and then compared. In the case of discrepancies, the final decision was made by a third reviewer (YZ). From each included study, we recorded: first author, publication year, original country where the study was conducted, number of participants, study design, age at enrollment in the study, outcome, and the effect estimate (95% CI).

### Statistical analysis

We extracted the association (OR or RR or HR) and their 95% CI or derived them by using data from the original studies (unadjusted RR = P_1_/P_0_, P_1_ indicates the incidence of the outcome of interest in the exposed group and P_0_ in the non-exposed group) [20]. The RRs were used as the common measure of association across studies. HRs were considered equivalent to RRs. The ORs were transformed into RRs using the formula RR = OR/ [(1 − P_0_) (P_0_ × OR)] [20]. We also compared the results applying the Miettinen test-based approach for calculating the variance of the natural logarithm of the RR (lnRR; variance lnRR = variance lnOR × (lnRR/lnOR)) [21].

Meta-analysis was performed using Stata V.12.0 software (Stata Corp, College Station, Texas, USA). We used the “metan” command in Stata to pool the lnRR. Forest plots were used to visually assess the RR estimates and corresponding 95% CIs across studies. In meta-analysis, the usual way of assessing whether a set of single studies is homogeneous is by means of the Q statistics. However, the Q statistic only informs meta-analysis about the presence versus the absence of heterogeneity, but does not report on the extent of such heterogeneity. Moreover, it has poor power to detect true heterogeneity among studies when the meta-analysis includes a small number of studies. Small studies have higher mean heterogeneity estimates than medium/large studies of the same meta-analysis [22]. I^2^ index is proposed to quantify the degree of heterogeneity and considered as a complement to the Q statistic, although it has the same problems of power with a small number of studies [23]. Therefore, we used the Q statistics (significance level of P < 0.10) and I^2^ statistics (> 50% was significantly inconsistent) to comprehensively evaluate between study heterogeneity [24]. Potential publication bias was assessed by using funnel plot and Begg’s and Egger’s tests, and P < 0.05 was considered significant.

## Results

### Studies selected and their characteristics

**Fig. 1:**
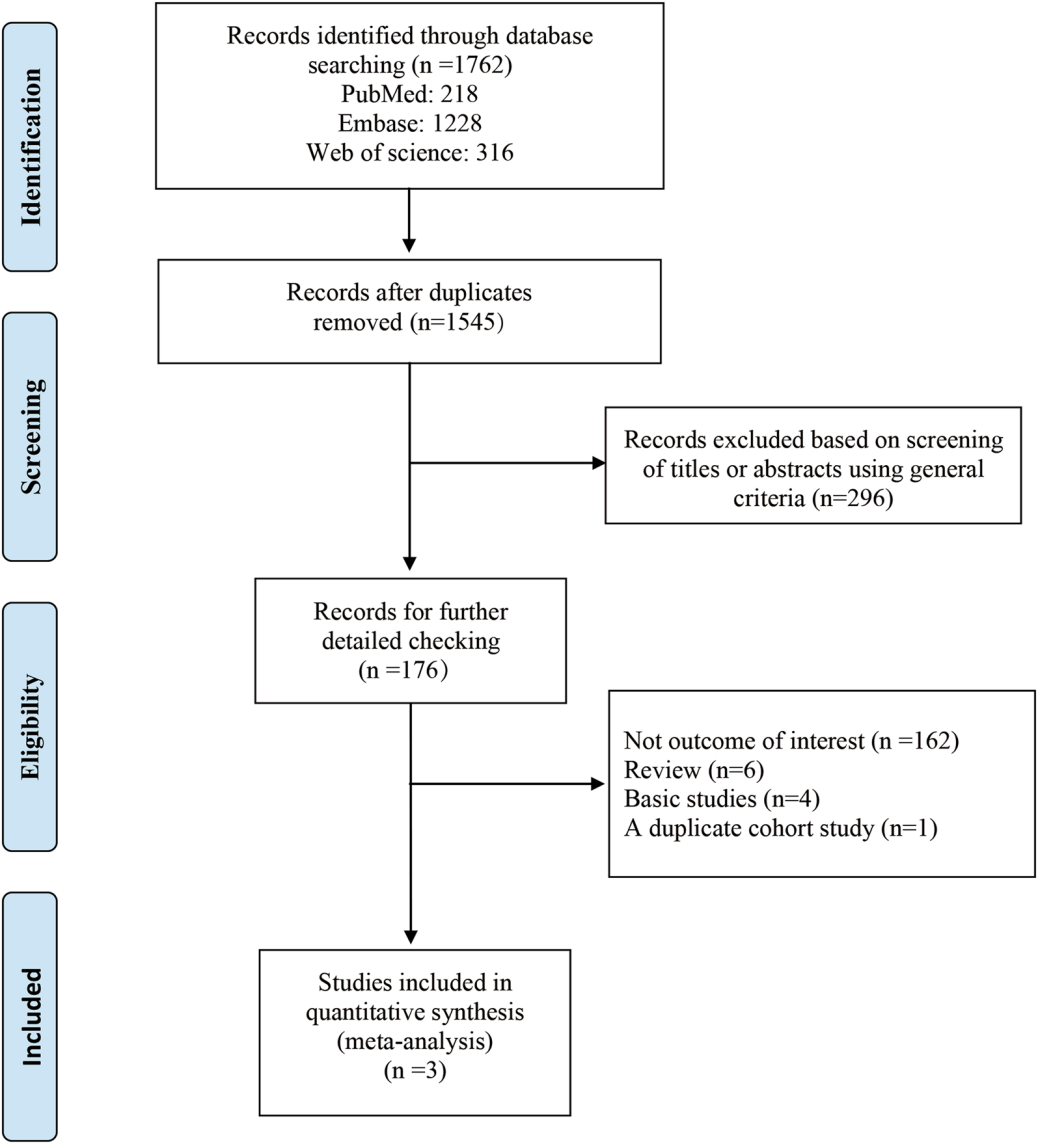
The flow diagram of identifying relevant studies

A total of 1762 studies were identified. Of them, 217 were excluded for duplicates and 1369 were excluded after screening the titles and abstracts, leaving 176 studies for full-text review. Two articles were published by the same authors in the same year and reported the results from the same database, but for different age groups (no age limit [14] vs  ≤ 18 years old [15]). To avoid duplicate inclusion of data, we selected only the more complete article [14]. Finally, 3 articles fulfilled the eligibility criteria and were included in this meta-analysis (Fig. 1) [14, 16, 18].

All selected studies were cohort studies and assessed by NOS and their score ranged from 6 to 8, suggesting that the methodological quality was acceptable. The main characteristics of studies are summarized in Table 1; more details about studies are shown in Additional file 2: Table S2.

**Table 1:** Characteristics of studies included in meta-analysis (n = 3)

Study	Published year	Country	No. of subjects	Study design	Age at enrollment in the study (years)	Outcome	Measure of association (95% CI)	NOS
Huang et al. [14]	2021	China (Taiwan)	173,720	A real-world, population-based cohort study	No age limit	Asthma	aHR 1.81 (1.74–1.88)	7
Kiefte-de Jong et al. [18]	2011	Netherlands	4651	A longitudinal birth cohort	Newborns	Wheezing	OR 1.10 (0.96–1.25)	7
Leander et al. [16]	2009	Sweden	290	A longitudinal population study	No age limit	Asthma	RR 8.24 (3.80–17.83)	6

#### Quantitative synthesis

**Fig. 2:**
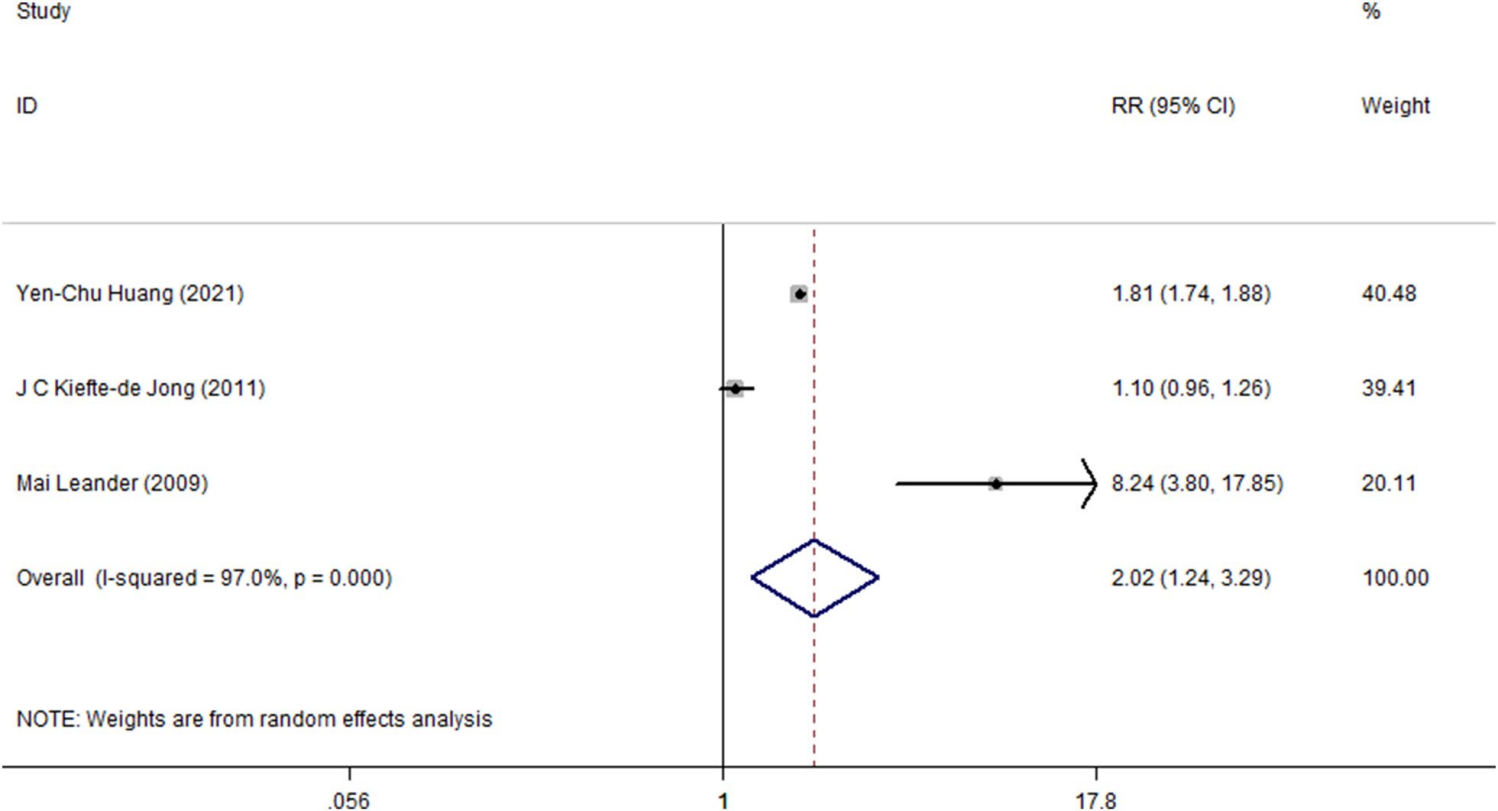
Forest plot of the overall association between constipation and the subsequent development of wheezing/asthma

The study by Leander et al. [16] did not provide RR between constipation and asthma. We could only calculate unadjusted RR (8.24, 95% CI = 3.80–17.83) using data from the original study (constipation in 25% of the 22 asthmatics vs 2% of the 268 non-asthmatics) by SPSS V.18.0. Significant heterogeneity among studies was present (I^2^ = 97.0%, P < 0.001), and thus a random-effects model was used. The combined results demonstrated that constipation was associated with increased risk of wheezing/asthma (RR = 2.02, 95% CI = 1.24 to 3.29, P < 0.01; Fig. 2).

#### Sensitivity and publication bias analysis

**Fig. 3:**
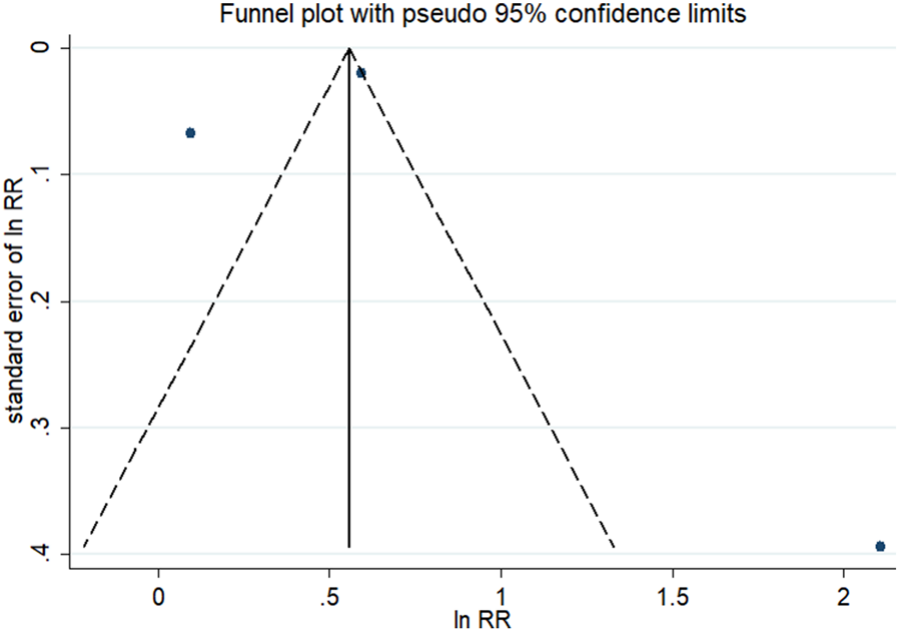
Funnel plot of publication bias for the association between constipation and the subsequent development of wheezing/asthma. The horizontal axis represents lnRR and the vertical axis means the SE of lnRR. Vertical line and sloping lines in funnel plot represent summary RR and expected 95% CI for a given SE, respectively

The funnel plot was used to evaluate publication bias, and there was no obvious asymmetry (Fig. 3). Furthermore, no significant publication bias was detected by Begg’s and Egger’s test (P > 0.05).

## Discussion

**Fig. 4:**
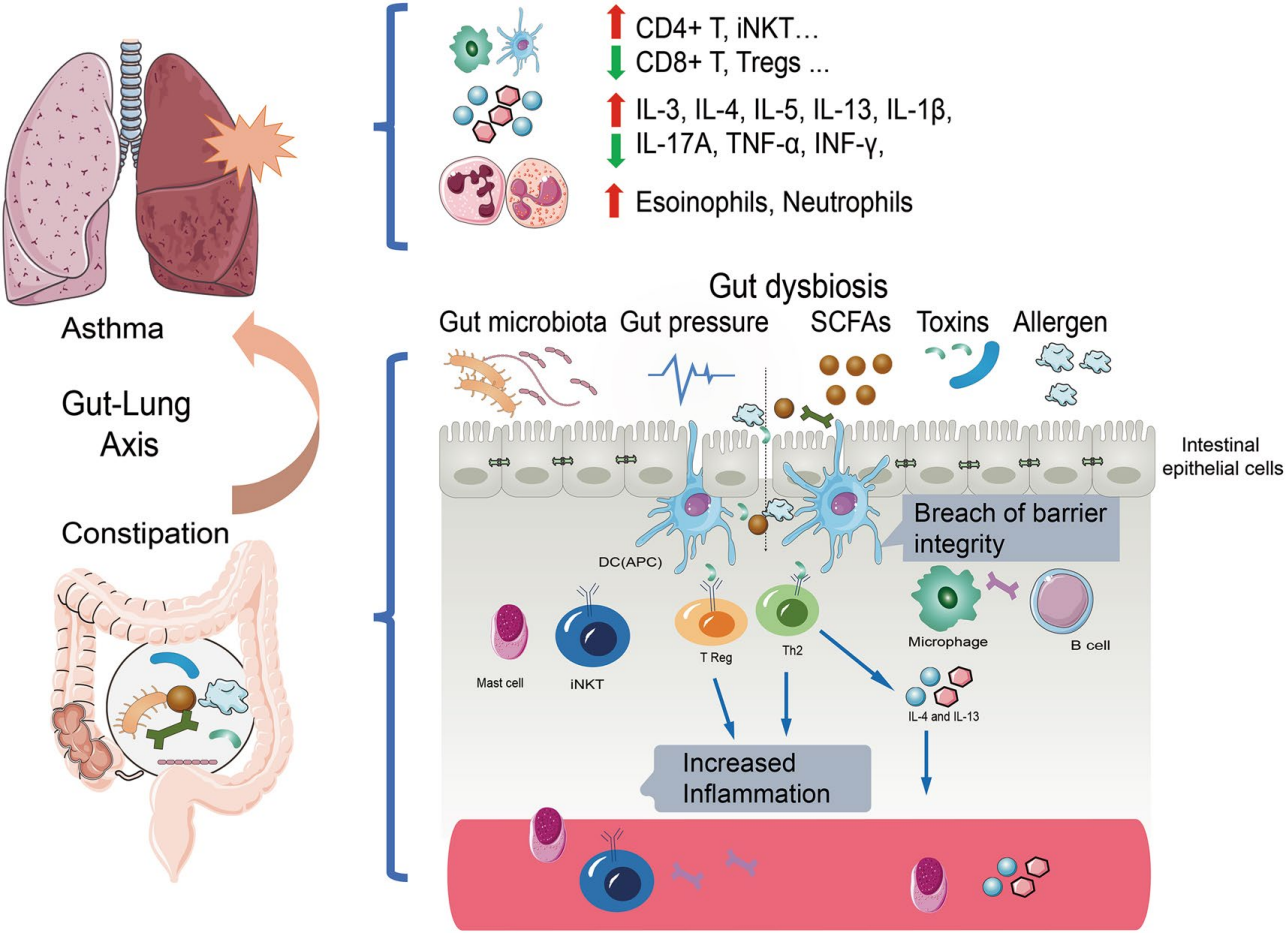
The role of gut-lung axis between constipation and asthma. Long term constipation causes imbalance of gut microbiota and gut pressure and the enrichment of toxins and allergen. The condition of gut dysbiosis leading to the dysregulation of gut inflammation and the defect of the intestinal epithelial barriers, resulting in leaky gut and allow penetration of microorganisms and toxins into systemic circulation, thereby activating Th2 immune responses and affects the development and function of Treg cells, eventually contributing to airway inflammation through the blood and lymphatic system

**Fig. 5:**
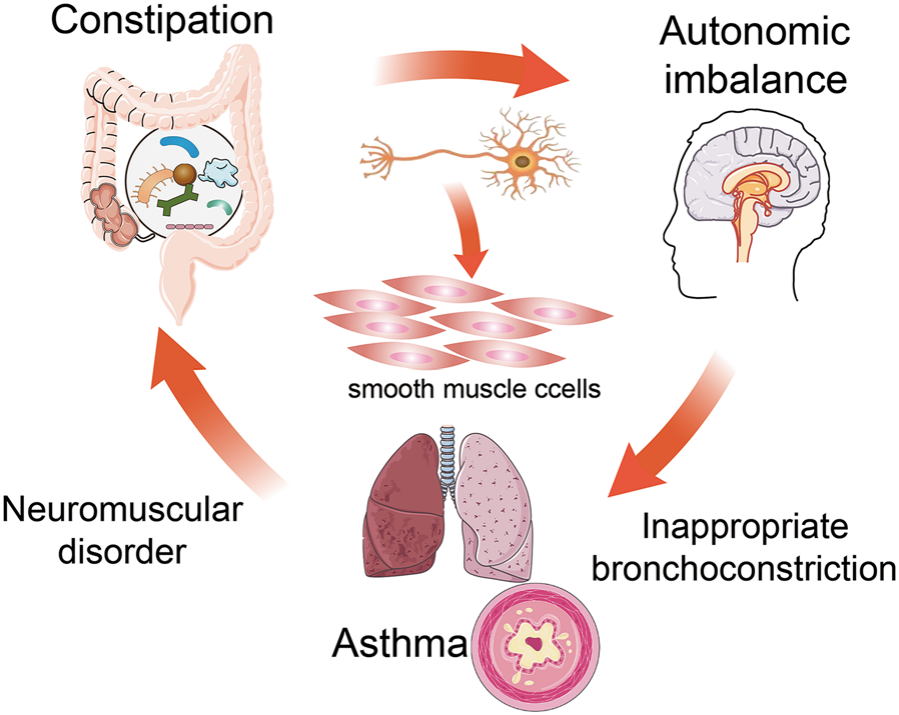
The expression of constipation might reflect an autonomic imbalance. Autonomic imbalance might also result to inappropriate bronchoconstriction as a response to an inhaled stimulus. Therefore, asthma and constipation may represent subsets of the same entity, which could be a primary neuromuscular disorder producing respiratory, gastrointestinal symptoms

The present meta-analysis on 3 studies provides considerable evidence indicating the positive association between constipation and the development of wheezing/asthma. Recently, a growing number of studies have been conducted to explore the association between constipation and the development of subsequent wheezing and asthma [14–16, 18]. However, the results are inconclusive. Therefore, it is critical to include all eligible studies and to assess the overall association. In this meta-analysis, the combined results demonstrated the association between constipation and the development of wheezing/asthma. Two potential biological mechanisms might underlie this association. (1) The condition of gut dysbiosis and lower concentrations of short-chain fatty acids (SCFAs) in the bowel decreases the production of butyrate and propionate, leading to the dysregulation of gut inflammation and the defect of the intestinal epithelial barriers, resulting in leaky gut and penetration of microorganisms and toxins into systemic circulation, thereby activating Th2 immune responses, eventually contributing to airway inflammation [14] (Fig. 4). (2) Constipation might reflect an autonomic imbalance [25], perhaps stemming from disturbed hypothalamic function [26]. A generalized autonomic imbalance might also result to inappropriate bronchoconstriction as a response to an inhaled stimulus [27]. Therefore, asthma and constipation may represent subsets of the same entity. This entity could be a primary neuromuscular disorder producing both respiratory and gastrointestinal symptoms in a relatively large proportion of patients (Fig. 5).

Therapeutic managements of constipation are increasingly incorporating prebiotics, probiotics or synbiotics with a view to regulating the intestinal microflora and suppressing both allergic and autoimmune responses [28]. Probiotics/prebiotics/synbiotics appear to have promising therapeutic effects for improving symptoms in people with asthma and allergies [29–31]. However, some mechanisms underlying intestinal microflora mediated regulation of immune responses being described are bacterial strain or metabolite specific, and should not be extrapolated to other probiotics or prebiotics. Additionally, the timing of intervention seems to be important, with potentially the greatest effects being observed early in life [28].

Several limitations should also be considered when interpreting our results: (i) the number of studies and subjects included in the present meta-analysis was relatively small. The limited number of included articles (n = 3) and great difference in sample size among studies might affect the representativeness of results from this meta-analysis. The study by Huang et al. only included participants from Taiwan, represented 97–98% of the total numbers. We could not investigate the influence of this single study on the overall risk estimate by excluding it as only 2 studies remained should not result in any model tests of significance. We will re-analysis and validate the risk identified in the current meta-analysis when more relevant studies are available. (ii) The diagnosis of wheezing/asthma was not always provided by a physician, this might affect the accuracy of the diagnosis and create a bias; Even if the diagnosis was made by a physician, it is not clear whether the physician was blind to the presence or absence of constipation. (iii) The large Taiwan study used constipation that was included in the health insurance database while the other 2 studies used questionnaire data. It is probable that constipation in the health database could be more severe/significant that that reported on questionnaires. Moreover, we could not confirm the accuracy of information obtained from questionnaire. (iv) It is commonly thought that asthma is a multi-factorial disease resulting from complex interactions between genetic predisposition and environmental factors, so the association of constipation with asthma would be affected by ethnic origin. However, we were not able to evaluate interaction effect of genetic backgrounds by constipation because there were only 1 study in Asians and 2 studies in European. In addition, although the results of some studies included in our meta-analysis were adjusted for some factors which might alter the association between constipation and wheezing/asthma risk, there were still other potential confounding factors. For example, smoking may be a confounding factor, which has been defined as a pivotal risk factor for constipation [32] and the development of asthma [33]. (v) NOS is a commonly used tool for quality assessment of non-randomized studies included in a systematic reviewer and/or analysis, however, using it may be controversial as the summary scores involve inherent weight of component items. No matter estimated effect varies with the quality score or not, the analyst can skip the quality-score analysis and go straight to the quality-component analysis to find out which components are responsible for the variation or avoid the risk of misleading conclusions, therefore, some researchers think that quality-score analysis may be superfluous [34]. (vi) Some data were not presented in the paper and were not obtained from the original researchers, so we could only derive unadjusted data using formulas or use the data obtained from another similar study, which may lead to biased results. (vii) Potential publication bias is also a concern. Although we did not observe apparent publication bias by statistical tests, it was still difficult to completely rule out this problem because there were not enough studies to detect it adequately.

## Conclusions

In conclusion, the current meta-analysis indicates that constipation may be associated with subsequent development of wheezing/asthma. In view of these limitations, more large-size prospective studies that adequately address concerns for potential confounding factors are required to validate the risk identified in the current meta-analysis.

## Supplementary Information

**Additional file 1.** The search strategies for PubMed, Embase and Web of Science.

**Additional file 2: Table S2. **The quality of each cohort study according to the Newcastle-Ottawa Scale (NOS) manual (n=3).

## Data Availability

The datasets used and analyzed during the current study can available from the corresponding author on reasonable request.

## Abbreviations

ICSs: Inhaled corticosteroids
NOS: Newcastle–Ottawa Scale
PRISMA: Preferred Reporting Items for Systematic Reviews and Meta-Analyses
RR: Relative risk
SCFAs: Short-chain fatty acids
SD: Standard deviation
