# Supplementary material for: Association between constipation and the development of asthma: a meta-analysis
Source: Allergy Asthma Clin Immunol. 2022 Aug 8;18:73. doi: 10.1186/s13223-022-00708-9 (PMC9358868; doi:10.1186/s13223-022-00708-9)
Supplement: Supplementary file 1 — Additional file 1. The search strategies for PubMed, Embase and Web of Science. [file 13223_2022_708_MOESM1_ESM.docx]

Key words

1. Constipation, functional fecal retention
2. asthma, wheezing

Pubmed 02/02/2022

| Search | Query | Results |
| --- | --- | --- |
| #1 | Search (Constipation) OR functional fecal retention | 33698 |
| #2 | Search (wheezing) OR asthma | 218356 |
| #3 | Search #1 AND #2 | 218 |

Emabase 02/02/2022

| No. | Query | Results |
| --- | --- | --- |
| #1 | 'constipation'/exp OR constipation | 110231 |
| #2 | 'functional fecal retention' | 36 |
| #3 | #1 OR #2 | 110233 |
| #4 | 'asthma' | 353579 |
| #5 | 'wheezing' | 34972 |
| #6 | #4 OR #5 | 368620 |
| #7 | #3 AND #6 | 1937 |
| #8 | #7 AND 'human'/de AND ('Article'/it OR 'Conference Abstract'/it OR 'Conference Paper'/it) | 1228 |

Web of Science 02/02/2022

| No. | Query | Results |
| --- | --- | --- |
| #1 | TS= (Constipation OR functional fecal retention) | 64918 |
| #2 | TS= (asthma OR wheezing) | 383147 |
| #3 | #1 AND #2 | 1323 |
| #4 | Document Types= ARTICLE | 316 |
