# Supplementary material for: Association between constipation and the development of asthma: a meta-analysis
Source: Allergy Asthma Clin Immunol. 2022 Aug 8;18:73. doi: 10.1186/s13223-022-00708-9 (PMC9358868; doi:10.1186/s13223-022-00708-9)
Supplement: Supplementary file 2 — Additional file 2: Table S2. The quality of each cohort study according to the Newcastle-Ottawa Scale (NOS) manual (n=3). [file 13223_2022_708_MOESM2_ESM.docx]

**Additional file 2: Table S2 The quality of each cohort study according to the Newcastle-Ottawa Scale (NOS) manual (n=3)**

| First author | Publication year | Selection | | | | Comparability | Outcome | | | Total score |
| --- | --- | --- | --- | --- | --- | --- | --- | --- | --- | --- |
|  |  | A | B | C | D | E | F | G | H |  |
| Yen-Chu Huang | 2021 | 1 | 1 | 1 | 1 | 1 | 1 | 1 |  | 7 |
| J C Kiefte-de Jong | 2011 | 1 | 1 | 1 | 1 | 1 | 1 | 1 |  | 7 |
| Mai Leander | 2009 | 1 | 1 |  | 1 | 1 | 1 | 1 |  | 6 |

**The NOS coding manual for cohort studiy**

**Selection**: A) Representativeness of the exposed cohort; B) Selection of the non-exposed cohort; C) Ascertainment of exposure; d) Demonstration that outcome of interest was not present at start of study. **Comparability. Outcome:** F) Assessment of outcome; G) Was follow-up long enough for outcomes to occur; H) Adequacy of follow up of cohorts
